# Editorial: Updates on inflammation in Parkinson's disease

**DOI:** 10.3389/fneur.2023.1138543

**Published:** 2023-01-20

**Authors:** Nicolas Dzamko, Marina Romero-Ramos, Avner Thaler

**Affiliations:** ^1^Faculty of Medicine and Health, The Charles Perkins Centre, School of Medical Sciences, University of Sydney, Camperdown, NSW, Australia; ^2^Department of Biomedicine, Danish Research Institute of Translational Neuroscience—DANDRITE, Aarhus University, Aarhus, Denmark; ^3^Laboratory of Early Markers of Neurodegeneration, Movement Disorder Unit, Tel-Aviv Faculty of Medicine, Tel-Aviv Medical Center, Sagol School of Neuroscience, Neurological Institute, Tel-Aviv University, Tel-Aviv, Israel

**Keywords:** Parkinson's disease, inflammation, innate immunity, alpha synuclein, COVID-19

Parkinson's disease (PD) is a progressive and disabling neurodegenerative disorder likely caused by a complex interplay of genetic and environmental factors. The immune system is one site of gene-environmental interactions, and its role in PD etiology is increasingly being recognized. Microglial response, the infiltration of peripheral immune cells, and neuroinflammation are prominent pathologies in PD (1), and increased pro-inflammatory cytokines have been measured in the blood, cerebral-spinal fluid (CSF), and brain tissue of PD patients (2, 3) and prodromal cohorts (4). In addition, changes in the peripheral blood immune cells have been also seen in prodromal (5, 6) and PD patients (7, 8). Several genes associated with PD risk have roles in immunity (9), and misfolded forms of the hallmark PD pathological protein, alpha-synuclein, can also activate the cellular immune system (10). Despite these strong associations, exactly how the immune system contributes to the pathogenesis and possibly even the onset of PD remains unknown.

In the current Research Topic, “U*pdates on Inflammation in Parkinson's Disease*,” we invited contributions that aimed to better understand the interplay of inflammation and PD.

Lerche et al. investigated the correlations between central (CSF) and peripheral (serum) cytokines, chemokines, and growth factors in a group of 453 idiopathic PD patients. Substantial sex differences were observed between males and females, and 25 and 38 percent of measured analytes showed a significant positive correlation between CSF and serum cytokines, respectively. This included the anti-inflammatory IL-4 for both sexes, the chemokines MIP1b for males and MCP1 for females, and the inflammatory cytokine IL-12 for females. However, stronger correlations for the clinical measures of disease progression were obtained from CSF cytokines rather than those from serum cytokines. This could indicate that unique inflammatory processes occur in the CNS and periphery of patients, and that CSF cytokines might better indicate the neuroinflammation associated with the progression of PD once the disease is manifest.

However, the relevance of the peripheral immune status was shown by Zheng et al. who specifically measured inflammatory markers not addressed by Lerche et al. including vascular cell adhesion molecule 1 (VCAM-1), soluble CD163, and the cell cycle regulating protein PRR14 (proline-rich protein 14) in serum from 100 PD patients and healthy controls. Higher levels of VCAM-1 and PRR14 were detected among patients with PD and were correlated with worsening PD symptoms.

The relevance of infections and associated immune responses as a cause of PD is explored by Zhang, who provided an insightful review into the possibility that SARS-CoV-2 infection may increase the risk of developing PD. Previous epidemiological studies and work in rodent models suggest that viral infection may predispose individuals to PD, potentially *via* triggering neuroinflammation. Zhang describes the symptomatic similarities between COVID-19 and PD, including case studies of acute parkinsonisms following COVID-19, and then proposes potential mechanisms by which SARS-COV2 infection may increase PD risk, for example by increasing the expression of alpha-synuclein protein.

Regarding alpha-synuclein, this protein continues to emerge as a potent regulator of both the innate and acquired immune systems. However, measuring alpha-synuclein is not without challenge. To facilitate new ways to quantify alpha-synuclein protein, Leupold et al. have developed a high-throughput flow cytometry-based assay for alpha-synuclein. By testing three commercially available antibodies they were able to identify a sensitive and specific antibody suitable for flow cytometry. Subsequent use of the optimized assay was undertaken to provide a cell-specific map for alpha-synuclein expression. This new tool could be particularly useful for understanding how alpha-synuclein expression relates to inflammation in PD patients.

Collectively these articles continue to build on evidence that inflammation is a key player in PD and an area that is still ripe for investigation into the causes of this enigmatic disease.

## Author contributions

All authors listed have made a substantial, direct, and intellectual contribution to the work and approved it for publication.
